# Trajectory-oriented Bayesian experiment design versus Fisher A-optimal design: an in depth comparison study

**DOI:** 10.1093/bioinformatics/bts377

**Published:** 2012-09-03

**Authors:** Patrick Weber, Andrei Kramer, Clemens Dingler, Nicole Radde

**Affiliations:** ^1^Institute for Systems Theory and Automatic Control, University of Stuttgart, Pfaffenwaldring 9, Stuttgart 70550, Germany

## Abstract

**Motivation:** Experiment design strategies for biomedical models with the purpose of parameter estimation or model discrimination are in the focus of intense research. Experimental limitations such as sparse and noisy data result in unidentifiable parameters and render-related design tasks challenging problems. Often, the temporal resolution of data is a limiting factor and the amount of possible experimental interventions is finite. To address this issue, we propose a Bayesian experiment design algorithm to minimize the prediction uncertainty for a given set of experiments and compare it to traditional A-optimal design.

**Results:** In an in depth numerical study involving an ordinary differential equation model of the *trans*-Golgi network with 12 partly non-identifiable parameters, we minimized the prediction uncertainty efficiently for predefined scenarios. The introduced method results in twice the prediction precision as the same amount of A-optimal designed experiments while introducing a useful stopping criterion. The simulation intensity of the algorithm's major design step is thereby reasonably affordable. Besides smaller variances in the predicted trajectories compared with Fisher design, we could also achieve smaller parameter posterior distribution entropies, rendering this method superior to A-optimal Fisher design also in the parameter space.

**Availability:** Necessary software/toolbox information are available in the supplementary material. The project script including example data can be downloaded from http://www.ist.uni-stuttgart.de/%7eweber/BayesFisher2012.

**Contact:** patrick.weber@ist.uni-stuttgart.de

**Supplementary Information:** Supplementary data are available at *Bioinformatics* online.

## 1 INTRODUCTION

Regulation models are preferably used to describe intra- and inter-cellular interactions of biomolecules. The Biomodel Database of the European Bioinformatics Institute comprises over 400 published and curated models in its repository, most of them being ordinary differential equation (ODE) models (Li *et al.*, 2010). The usefulness of a biomedical ODE model is often assessed through its capability to predict possible scenarios of interest. In order to calibrate a biological ODE model, expensive and time-consuming experiments are performed to gain the needed experimental training data. Despite continuously improving measurement methods, the data for most models remain scarce, resulting in practically non-identifiable parameters (Gutenkunst *et al.*, 2007). To support the learning process, several experiment design methods have been successfully developed including classical Fisher information matrix (FIM) design (Bandara *et al.*, 2009), Bayesian methods (Chaloner and Verdinelli, 1995; Kramer and Radde, 2010; Wilkinson, 2007) and optimization-based methods (Kreutz *et al.*, 2011). Claiming that ODE model predictions improve when reducing parameter uncertainty intervals, these methods design optimal experiments for parameter estimation (OED/PE). However, models can be used to make precise predictions despite sloppy parameters with large confidence intervals (Brown *et al.*, 2004; Klinke, 2009). This is rendering OED/PE an indirect method to improve predictions. Methods have been developed to directly address experiment design for better model predictions (Casey *et al.*, 2007; Vanlier *et al.*, 2012), which we here refer to as OED/MP. In this work, we also propose to go this direct way, by predefining sets of experimentally feasible prediction scenarios of interest. We use a Bayesian posterior inference method and predict experimentally feasible scenarios to purposively reduce uncertainty in the model trajectories. In addition, we define a reasonable stopping criterion to avoid further experimentation when the desired predictions meet a certain precision compared with the expected data quality. The method is successfully applied to reduce prediction uncertainty of an ODE model of secretory pathway control at the *trans*-Golgi network. In an intense comparison study, we were able to outperform A-optimal designed experiments in both prediction uncertainty and posterior distribution entropy, with affordable computational effort.

## 2 METHODS

### System

In this work, we address the problem of efficiently selecting experiments to improve the prediction capabilities of a given ODE model of the following form:
(1)
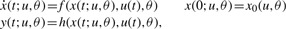

with state ℝ*x(t;u,θ)* ∈ ℝ_+_*^n_x_^*, input *u(t)∈* ℝ*_+_^n_u_^*, parameter *θ* ∈ ℝ*_+_^n_θ_^* and output *y(t;u,θ) ∈ ℝ_+_^n_y_^*. The vector field *f* is assumed to be continuously differentiable to guarantee the existence of a unique solution. For intracellular models, *f* is often derived by using chemical reaction kinetics. The output mapping *h* defines the measurable outputs as functions of model states. To include typical experimental pretreatments, we allow the initial conditions *x*_0_(*u, θ*) to depend on the system parameters *θ* and initial experimental influences *u*. The system parameter vector *θ* consists of positive rate coefficients, Michaelis Menten parameters or degradation and synthesis rates. Given positivity, these constants are assumed to be log_10_-transformed, *θ* = {*θ*_1_,...,*θ_nθ_*} = {log_10_(*k*_1_),...,log_10_(*k_nθ_*)}, to guarantee numerical stability in subsequently introduced numerical computations.

#### Experiments

The goal of improving the model prediction is restricted to a predefined set of experiments {*ℰ^i^*}_*i*=1_*^nℰ^*. In This Work, We Consider Each Prediction Scenario also a potential experiment. Each experiment index *i* is connected to an experimental setup. An experiment *ℰ^i^* is defined by an experimentally feasible input vector *u^i^* (*t*) and a set of measurable quantities *y^i^* = (*y*_1_^*i*^,..., *y_n_y^i^__^i^*). Typical experiments include for example initial changes of *u^i^*(*t*) at *t*_0_ describing vector overexpression or siRNA experiments. Also popular are specific changes at a predefined time instance that refer to culture medium changes or external stimuli. Each measurable quantity *y_j_^i^*, *j* = 1,..., *n_y_^i^* can be measured at one discrete time point *t_j_^i^* within the set 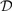 .

#### Data and likelihood function

We assume that the measurement process is prone to measurement noise, and we choose a log-normal error model here, in accordance with recent studies (Gassmann *et al.*, 2009; Kreutz *et al.*, 2007). Thus, whenever a measurement is performed, the true system is assumed to provide a data point of the form
(2)
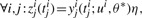

with 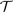 , θ^*^ denoting the true system parametrization and σ^*^ denoting the true error model parameter. For simplicity, we assume the same parameter *σ* for all outputs and experiments, which is no general restriction for our proposed method. We collect the data from experiment *i* in the set 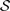 . Given this error model and assuming independence of all measured data points, the likelihood function for the system states
(3)
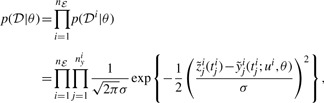

with the log transformations 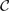 and 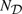 . The maximum-likelihood estimate (MLE) for the parameters is given by maximizing the likelihood function:
(4)
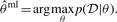


### 2.1 Trajectory-oriented Bayesian design

The objective function in the Bayesian framework is the posterior distribution 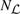 , which is a distribution over the parameters *θ* after having seen the data. Values of the posterior distribution can be calculated using Bayes' theorem:
(5)
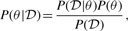

where *P*(*θ*), the prior distribution, reflects our prior assumptions about the parameters. *P*(*θ*) can be used to restrict the search space to regions of plausible values. 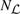 can be investigated through Markov chain Monte Carlo sampling, and we use an adaptive Metropolis algorithm for this purpose.

In the following, we denote the representative sample for the current posterior distribution {*θ*^*s*^: *s* = 1,...,*N*} or short {*θ*^*S*^}. Each sample step requires evaluation of the likelihood function, for which the ODE system has to be solved numerically. Thus, together with a sample {*θ*^*S*^}, we get automatically for each experiment *i* and each measurable output *y_j_^i^* a set of trajectories {*y_j_^i^* (*t; θ^s^*,*u^i^*): *s* = 1,...,*N*}. From those trajectories, we can estimate variances according to 
(6)
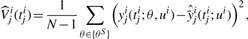

with estimated mean
(7)
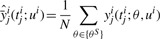

for each *i*, *j* and each time point 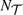 . In the first step, we determine the set of measurement times 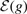 that maximize the expected variances in the trajectories
(8)
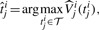

and collect the set of respective variance estimates in the sets 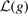 . These sets are the basis for our experiment selection procedure. At this point, we introduce a stopping criterion: measurement on *y_j_^i^* in experiment *i* is performed only if the respective variance 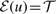 is still above a certain threshold, which we set to the pooled empirical variance estimate 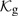 here. This means that we propose to measure only if we can still expect a decrease in 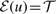 even when we take the precision 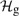 of the measurement process into account. To address this stopping criterion mathematically, we set 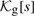 . The successor experiment *î* is selected by maximizing the sum of expected variances within each experiment
(9)
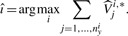

Once *î* has been determined, we suggest to measure at time instances 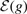 for which 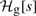 .

**Fig. 1.**
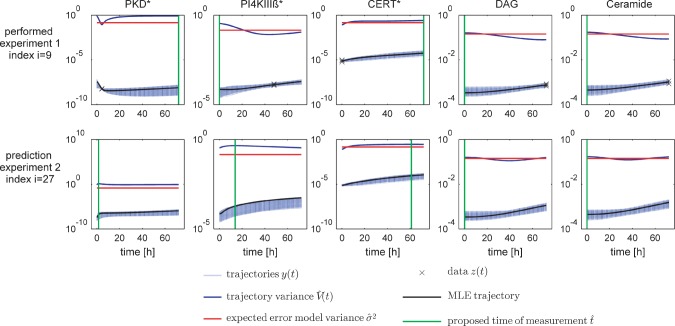
Predicted trajectories for the experiment outcome after an initial training experiment is performed. Trajectories are depicted together with the trajectories' empirical variance. The first row depicts the model fit for the initial experiment. The measurement time instances suggested by our design algorithm are depicted by vertical lines. The second row evinces the proposed measurements for the second successor experiment. For experimental inputs corresponding to experiment index *i*, we refer to Supplementary Table S3

To illustrate this approach, we refer to a fetch-ahead of our numerical study given in Figure 1, which shows the sets of trajectories {*y_j_^i^*(*t; θ^s^*, *u^i^*)} of the posterior sample {*θ*^*S*^ } for each measurable quantity *y_j_^i^* after a first experiment was performed (upper row), along with the proposed measurement time instances 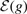 , which are indicated with vertical lines here, the estimated variances 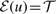 and the current values of the pooled measurement error estimates 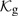 . The second line shows predictions subject to the current sample {*θ*^S^} for a different experiment. In this case, the algorithm would decide to measure all quantities *y_j_^i^* once again, since the stopping criterion is fulfilled for none of them.

### 2.2 FIM experiment design

To compare our proposed Bayesian experiment selection to a computationally inexpensive and well-established experiment design routine, we shortly introduce the well-known FIM based experiment design routine (Atkinson and Donev, 1992). The improvement of FIM optimal experiment design for time-series measurements in biomedical model discrimination (OED/MD), OED/PE or the combination of both disciplines is in the focus of recent publication series (Donckels *et al.*, 2012, 2009), rendering it a solid reference method. In order to apply the method, the FIM must be evaluated at the current MLE 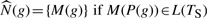 for the current index set *ℐ_p_* of all already performed experiments:
(10)
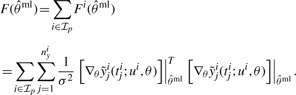

The superposition principle of the FIMs holds for experiments, outputs and measurement time points. In the next step, anticipatory update FIMs 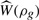 for all candidate experiments *i* = 1,..., *nϵ* and *j* = 1,..., *n_y_^i^* are evaluated at the current MLE. The update FIMs 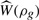 are added individually to the current FIM, forming the overall predicted FIMs 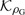 . A design criterion 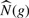 has to be applied to the update FIMs to decide upon the successor experiment for the current design step. Under the threat of non-identifiable parameters, we decided to apply the modified A-optimal criterion 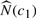 to completely avoid the inversion of close to singular FIMs. For fairness of comparison, we need to have the same amount of overall measurements *m* for the next experiment, which is determined by the Bayesian design. If *m* outputs are to be measured at yet unknown *t_j_^i^*, these are chosen according to
(11)
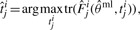

(12)
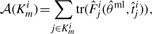

(13)
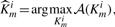

(14)
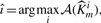

where *K_m_^i^* denotes the index subset of the set of all measurable outputs *y_j_^i^* with *m* elements. The successor output set 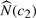 and time points 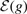 contain all needed information to plan the experiment. We note that other FIM design criteria such as minimizing the determinant of the inverse matrix are more involved computationally, since the superposition principle does not hold any more and maximization cannot be decoupled any longer.

## 3 DESIGN OF THE STUDY

### 3.1 A model for secretion control at the *trans*-Golgi network

**Fig. 2.**
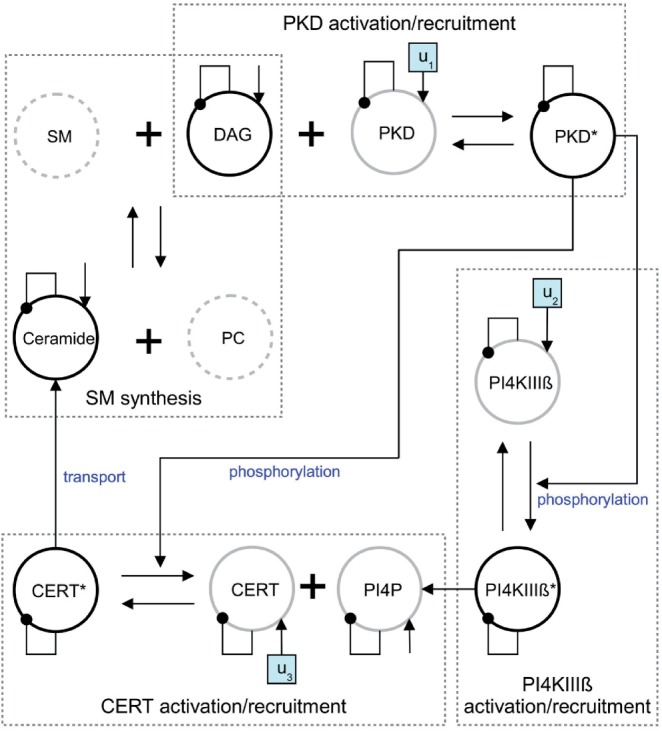
Model for secretion control at the *trans*-Golgi network. Grey nodes denote latent variables, black nodes measurable species. Active species are denoted with an asterisk. Self-loops describe linear degradation terms. Dashed boxes depict turnover reactions. The inputs ‘u’ indicate experimentally possible perturbations of synthesis rates. Blue text explains biological processes depicted by corresponding edge. SM and PC are not contained in the model but shown for completeness of the SM synthesis reaction

Protein secretion in mammalian cells is a highly regulated process, in which the regulation of the formation of transport vesicles at the *trans*-Golgi network plays a crucial role. Understanding these regulatory processes is for example important for further optimization of producer cell lines, where it has been shown that for high messenger RNA (mRNA) copy numbers, there are bottlenecks further downstream, and one example is the *trans*-Golgi network (Becker *et al.*, 2008). Vesicle formation is mediated by a network of interacting proteins and lipids, involving the protein kinase D (PKD), the ceramide tranfer protein CERT, the lipid kinase PI4KIIIβ, ceramide and diacylglycerol (DAG). Figure 2 shows a scheme of our regulatory network model. DAG is located in the Golgi membrane and recruits and thereby activates PKD (Bard and Malhotra, 2006). Active PKD has a dual effect on CERT transport activity: on the one hand, it directly phosphorylates and thereby deactivates CERT (Fugmann *et al.*, 2007), on the other hand, it activates CERT indirectly through activating PI4KIIIβ, which triggers synthesis of phosphatidyl-inositol-4-phosphate (PI4P). PI4P enables binding of CERT to the TGN and release of ceramide (Fugmann *et al.*, 2007). The feedback loop between CERT and PKD is closed by conversion of phosphatidylcholine (PC) and ceramide to sphingomyelin (SM) at the Golgi, where DAG occurs as a byproduct. Both PKD and DAG have also been shown to play a direct role in vesicle formation (Bard and Malhotra, 2006; Hausser *et al.*, 2005). Although the main interactions are known, the kinetics of this system has not yet been investigated quantitatively, and there are currently no appropriate experimental data available for model fitting. Thus, we use artificial data here.

A differential equation model for the regulation process was established which involves 9 molecular species, 17 chemical reactions, 12 interaction parameters and 17 further parameters such as basal production and degradation rates. Up to five model outputs are measurable upon request, namely active PKD (PKD^*^), active PI4KIIIβ (PI4KIIIβ^*^), active CERT (CERT^*^), overall DAG and overall ceramide. The four remaining model status variables are defined as latent variables. Equations and parameter values are listed in Supplementary Appendix.

### 3.2 Experiment design testing

In order to test our newly suggested algorithm, we performed a biologically motivated *in silico* study. Our Bayesian and a classical A-optimal design routine are allowed to make suggestions from a set of 27 experiments, which describe all possible perturbation permutations of the three species that can experimentally be accessed through overexpression or siRNA silencing, namely PKD, CERT and PI4KIIIβ. The valid experiment time horizon has been set to *t*_end_ = 72h, which is a realistic time frame for a cell culture experiment with proliferation and confluence effects. In the case of silencing, the respective basal expression rate is damped by a factor of 10, while in the vector overexpression case, the basal rate is 10-fold higher. These input perturbation parameter values for siRNA silencing and vector overexpression are experimentally motivated by typical suppression rates (Pillai *et al.*, 2005). Although the Bayesian approach can in principle incorporate input uncertainty, this is not considered in this comparison study to avoid shifting the focus.

We assume all 12 interaction parameters to be initially unknown and choose a bounded log uniform prior supporting four orders of magnitude for each of them. Parameters are estimated and sampled in logarithmic space, while basal production and degradation rates are set to biologically plausible values (Eden *et al.*, 2011). To account for limitations in the quantity of sampling time instances in protein quantification (e.g. western blotting: 18 lanes per gel), both algorithms were allowed to demand for a maximum of one data triplet at one sampling time instance for each output. Thus, if for example data for all five outputs are requested this would result in 15 data points in total. The requested data are generated by the unknown true system and noise corrupted using a log-normal error model with σ^*^ = 0.15. The exercise is stopped as soon as one algorithm trained the model in a way that it fulfills our stopping criterion. Details on the choice of the joint initial experiment are given in the Supplementary Material. To make the study more realistic, we estimate the variance 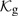 in each simulation scenario using a pooled variance estimate. In order to compare the two algorithms, the 12-dimensional parameter posterior distributions have been estimated through Markov chain Monte Carlo (MCMC) sampling after each newly obtained dataset. For their computation, the sampling algorithms of the MCMC toolbox (Haario *et al.*, 2006) have been combined with the fast ODE integrators of the SBtoolbox2 (Schmidt and Jirstrand, 2006) and the parallel computing capabilities of Matlab. Model prediction and entropy estimation in the following sections are based on these posterior distributions. For the prediction of our proposed Bayesian method, 1000 parameters were drawn from the posterior distribution according to the description in ‘Methods’ section, and all 27 experiments are simulated. The time horizon of 72 h was divided into 105 equidistant discrete sampling times, leaving realistic 40 min of reaction time for an experimentalist between two subsequent wet lab sample preparation procedures. The whole design exercise has been repeated five times for each algorithm.

## 4 RESULTS

### 4.1 Bayesian experiment design results in twice the prediction quality than A-optimal design

**Fig. 3.**
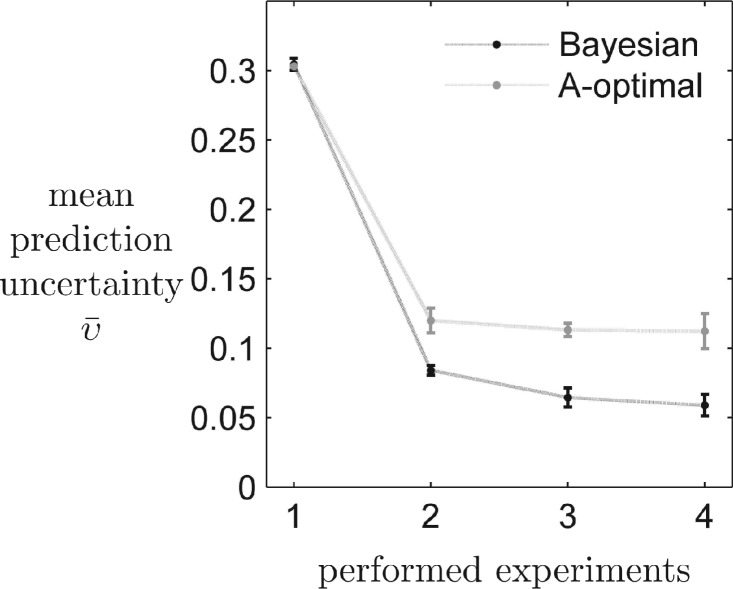
Mean expected prediction uncertainty for all 27 experiments after the model has been trained with up to four experiments chosen by the Bayesian or the A-optimal design criterion. We additionally averaged over five different runs

Figure 3 depicts the expected uncertainty of the predictions, represented here as the overall mean of all expected variances 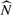 after being trained with experiments selected by the competing algorithms. We additionally averaged over five runs. After each new training experiment, a prediction for all experiments has been performed. The stopping criterion was reached by the Bayesian routine after four experiments in all five runs, while requesting a total of 12 data points. This means that we expect the trained model to be able to make predictions for all 27 experiments in which all trajectory variances are below the threshold.

The evolution of the mean trajectory variance 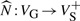 , averaged over all 27 experiments, outputs and time points, is depicted in Figure 3. It reaches a value of 0.06 after four experiments for the Bayesian design, making the model prediction twice as good as the A-optimal trained model with a value of 0.12. After the terminal experiment 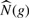 (see Supplementary Appendix).

### 4.2 Bayesian experiment design gains more information about parameters than A-optimal design

**Fig. 4.**
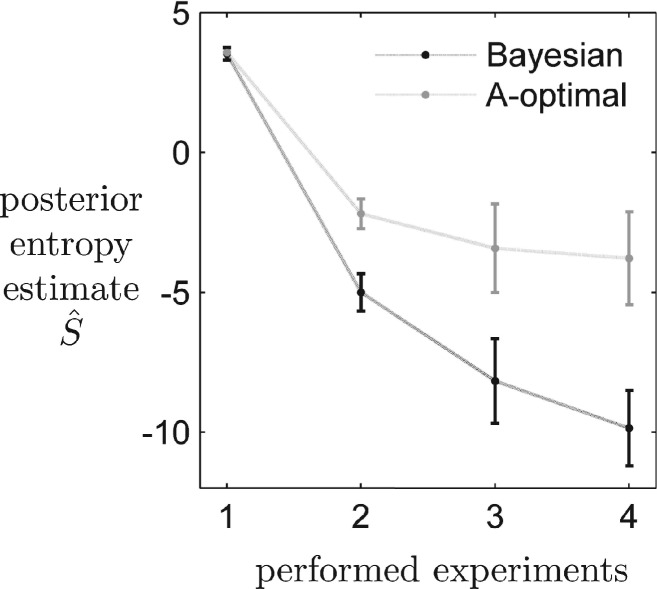
Mean posterior distribution entropy estimates after the model has been trained with up to four experiments chosen by the Bayesian or the A-optimal design criterion, respectively. Five runs of the overall experiment design exercise have been performed

Since A-optimal design reduces the overall spread of the posterior distribution, while the proposed method reduces the observable trajectory variance, we should also consider the posterior size in a fair comparison. The entropy *S* reflects the spread of a probability distribution regardless of shape complexity. Therefore, we include a numerical approximation of the entropy given by
(15)
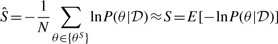

in this analysis as a measure for the information content of the posterior sample of the parameters. Figure 4 depicts the evolution of the Shannon entropy estimated from the posterior samples using a parallel multivariate kernel density estimator. Our prediction-based Bayesian design routine achieved an average entropy estimate of –10 nats, which is 8 nats lower than the A-optimal experiments with an average value of –2 nats. Our design is superior to A-optimal design already after the second experiment.

**Fig. 5.**
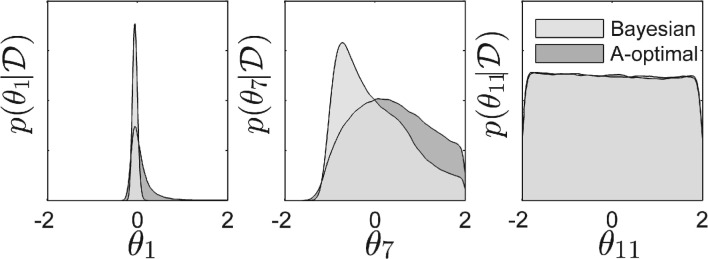
Estimated marginal distributions of three parameters of the posterior sample after the model has been trained with four experiments chosen by the Bayesian or the A-optimal design criterion. *Y*-axis limits denote the boundaries of the log uniform prior

As entropy is an abstract measure, an additional visual insight is given by Figure 5, which shows a comparison of the marginal posterior distributions for three parameters of one exemplary design run. We have exemplary chosen a parameter that is expected to be very well identifiable after the design exercise with four successive experiments, one that is vaguely identifiable and one that is expected to be not identifiable at all. Axis ranges have been set equal to the prior ranges. We have also visually inspected all marginal distributions of five different design runs and categorized the established parameter boundaries in Table 1. Although this is not an established quantitative measure as, e.g. entropy, it is a good visualization for the differences of the two designs in the parameter space.

**Table 1.** Qualitative properties of the model parameters

Algorithm	Two boundaries	One boundary	Prior bounded
Bayes design	5.6 ± 0.5	4.2 ± 0.7	2.2 ± 0.4
A-optimal design	4.6 ± 1.4	3.6 ± 1	3.8 ± 1.2

Average number of parameter boundaries distinct from the initially set prior boundaries established after the fourth experiment in five runs. The categories two boundaries, one boundary and prior bounded parameters are determined by visually examining the marginal distributions (Figure 5).

In our example, the parameter *θ*_1_ describes recruitment and thereby activation of PKD by DAG. The flux for this reaction depends in our model linearly on both variables. Furthermore, PKDa and DAG are both measurable, such that it is intuitive that *θ*_1_ becomes identifiable. In the five runs, the Bayesian design routine established an average of one more double bounded parameters such as *θ*_1_, than A-optimal design. Double bounded parameters with tight confidence intervals are most useful to draw biological conclusions, as they restrict model fluxes. As each flux stands for a different effect (e.g. transport or activation), the relative importance of these effects becomes comparable.

The parameter *θ*_7_ describes recruitment of CERT to the TGN through PI4P and its activation. Although CERT can be disturbed and is also directly measurable, both are not the case for PI4P, and so the experiments are less informative for this parameter. Still, an average of 0.6 more one-sided parameter boundaries such as the lower bound of *θ*_7_ are identified by the proposed method. With the same line of arguments, upper or lower bounded parameters are useful to identify, e.g. a tendency towards higher or lower influence of a modelled effect.

Finally, *θ*_11_ describes conversion of DAG to ceramide. Although both DAG and ceramide are observables, this parameter is hardly identifiable, since the flux of this reaction is small in our model and both variables cannot be directly perturbed. Bayesian design ended up with an average of 1.6 less of these uninformative parameters. An example plot of all 12 marginal distributions of the parameter vector is given in Supplementary Figure S2. Summarizing it can be said that the Bayesian design enables a more detailed interpretation of the model with the same amount of data measured by more efficiently restricting the models parameter values.

### 4.3 Bayesian design makes more consistent decisions

**Fig. 6.**
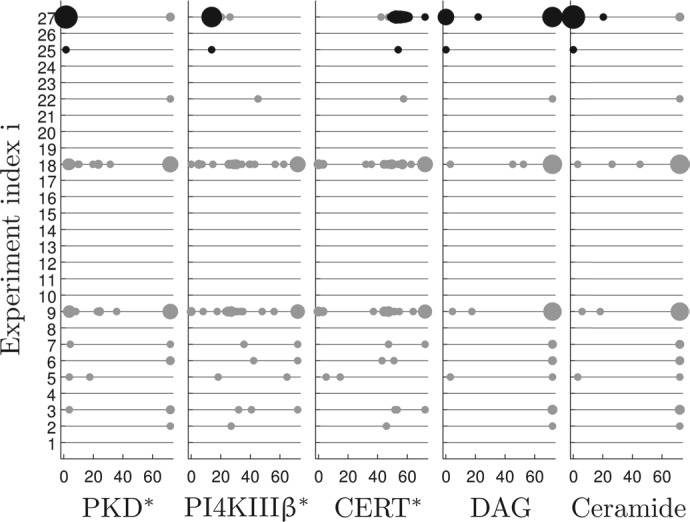
Repeated decision making (*n* = 100) of the Bayesian (black) and the A-optimal Fisher (grey) experiment design. Big spots indicate more frequently chosen combinations of experiments and sampling times

Figures 3 and 4 indicate that the highest incremental improvement in predictions and entropy is achieved after the second experiment. We used this particular design step to further investigate differences in decision making of the competing algorithms. We tested two potential weak points of each method. Fisher design is assumed to be highly dependent on the MLE. Therefore, we repeated the second design step 100 times, while each time re-estimating the MLE. Bayesian design is limited by the amount of representative posterior trajectories that can be simulated for decision making; therefore, we redraw 100 times the *N* = 1000 prediction trajectories from the posterior distribution. The statistic for the proposal of the second experiment is depicted in Figure 6. Trajectory-based Bayesian design prefers (beside one single outlier) experiment 27, overexpression of all three species, while merely altering in sampling time. A-optimal decisions are mostly bi-modal between experiments 9 and 18, in which PI4KIIIβ and CERT are overexpressed, while about 15% of the decisions are scattered over various experiments and time points. It is clearly visible that the suggestions of the Bayesian design are much more consistent.

### 4.4 Computational effort

The main computational effort of this study stems from the sampling of the posterior distribution, which serves as a common criterion to compare the two different methods. This involved over a quarter of a billion ODE integrations and a detailed Markov chain convergence analysis. Parallel Markov chains have been merged for each posterior distribution estimate containing at least 3.5 million sample points each. Not converged sub-chains have been discharged. Note that experiments are typically scheduled on a weekly basis, so that differences in computation time might not be crucial. The posterior sampling has to be performed only after new data is received from the wet lab. For a fair comparison of the computational effort of the two design routines, we focus on the number of necessary ODE integrations needed for the actual design or proposal steps which are, respectively, used to assess new potential experiments. The calculation of the A-optimality of a candidate experiment with a 12×12 FIM (simple finite differences) requires 48 model simulations; the Bayesian concept required 1000 simulations per prediction, rendering it 20 times more simulation intensive than the Fisher design in this example. Still, any state of the art Desktop PC should be able to assess 100–1000 experiments per hour using the Bayesian method.

## 5 DISCUSSION AND CONCLUSION

In this work, we introduced a trajectory-based Bayesian experiment design approach and compared this with A-optimal Fisher design for a regulatory network of transport vesicle formation at the *trans*-Golgi network in mammalian cells with 12 parameters. In this example, which contains several sloppy parameters, Bayesian design clearly outperformed Fisher design with respect to reducing uncertainty in predictions and also in the parameter space.

Our results demonstrate that design criteria that rely on local approximations of the posterior distribution are not suited in case of sloppy or non-identifiable parameters. Fisher design, for example, assumes that the posterior distribution can be approximated by a multivariate Gaussian about its maximum. In our study, we could further identify vulnerable dependence of FIM design decision making to the obviously non-reliable MLE. Since the appearance of sloppy parameters is ubiquitous for intracellular regulation networks, we strongly encourage to use and further develop methods that are based on output or trajectory optimization rather than parameter identification.

One of the reviewers brought up the important point of model errors, which leads to further discrepancies between model simulations and ‘real’ observations. This study uses artificial data that are generated by the model under consideration, which has of course the advantage that all true parameters are known and results are controllable. However, we are aware that this simplifies analyses a lot compared with more realistic settings with real experimental data. For example, if the model is erroneous and does not really capture the dynamics of the underlying processes, this might lead to likelihood functions for different datasets that are not consistent with respect to the model and favour different regions in the parameter space, which complicates the PE step and thus also the whole experiment design method. This fact has for sure to be taken into account when working with real data and can for example be addressed by a design strategy that is optimized to discriminate between different model hypotheses, which is one of the main issues for our future work in this project.

When summarizing our numerical results, we became aware of a very recent study on prediction-based experiment design (Vanlier *et al.*, 2012). The authors focus on posterior predictive distributions, which assign probabilities to new simulation scenarios. Although this is an elegant approach in general, calculating this distribution requires the evaluation of further integrals and thus becomes rapidly computationally expensive for high-dimensional design spaces, e.g. if multiple candidate measurements are planned within a single experiment.

Overall, model-based experiment design that optimizes prediction uncertainty is a challenging new field, which is very suitable especially for intracellular network models with sparse data and should further be investigated.

## 6 AUTHOR CONTRIBUTIONS

P.W. and A.K. developed the trajectory-based Bayesian design algorithm. P.W. implemented the algorithm and created the results with support from A.K. C.D. created results for the Fisher design. P.W., A.K. and N.R. developed the model and wrote the manuscript. All authors read and approved the final manuscript.
